# Analysis of TcdB Proteins within the Hypervirulent Clade 2 Reveals an Impact of RhoA Glucosylation on Clostridium difficile Proinflammatory Activities

**DOI:** 10.1128/IAI.01291-15

**Published:** 2016-02-24

**Authors:** Carlos Quesada-Gómez, Diana López-Ureña, Nicole Chumbler, Heather K. Kroh, Carolina Castro-Peña, César Rodríguez, Josué Orozco-Aguilar, Sara González-Camacho, Alexandra Rucavado, Caterina Guzmán-Verri, Trevor D. Lawley, D. Borden Lacy, Esteban Chaves-Olarte

**Affiliations:** a Facultad de Microbiología and Centro de Investigación en Enfermedades Tropicales, Universidad de Costa Rica, San José, Costa Rica; b Department of Pathology, Microbiology, and Immunology, Vanderbilt University School of Medicine, Nashville, Tennessee, USA; c Facultad de Farmacia, Universidad de Costa Rica, San José, Costa Rica; d Laboratorio de Ensayos Biológicos, Escuela de Medicina, Universidad de Costa Rica, San José, Costa Rica; e Instituto Clodomiro Picado, Facultad de Microbiología, Universidad de Costa Rica, San José, Costa Rica; f Programa de Investigación en Enfermedades Tropicales, Escuela de Medicina Veterinaria, Universidad Nacional, Heredia, Costa Rica; g Host-Microbiota Interactions Laboratory, Wellcome Trust Sanger Institute, Hinxton, United Kingdom; h The Veterans Affairs Tennessee Valley Healthcare System, Nashville, Tennessee, USA

## Abstract

Clostridium difficile strains within the hypervirulent clade 2 are responsible for nosocomial outbreaks worldwide. The increased pathogenic potential of these strains has been attributed to several factors but is still poorly understood. During a C. difficile outbreak, a strain from this clade was found to induce a variant cytopathic effect (CPE), different from the canonical arborizing CPE. This strain (NAP1_V_) belongs to the NAP1 genotype but to a ribotype different from the epidemic NAP1/RT027 strain. NAP1_V_ and NAP1 share some properties, including the overproduction of toxins, the binary toxin, and mutations in *tcdC*. NAP1_V_ is not resistant to fluoroquinolones, however. A comparative analysis of TcdB proteins from NAP1/RT027 and NAP1_V_ strains indicated that both target Rac, Cdc42, Rap, and R-Ras but only the former glucosylates RhoA. Thus, TcdB from hypervirulent clade 2 strains possesses an extended substrate profile, and RhoA is crucial for the type of CPE induced. Sequence comparison and structural modeling revealed that TcdB_NAP1_ and TcdB_NAP1V_ share the receptor-binding and autoprocessing activities but vary in the glucosyltransferase domain, consistent with the different substrate profile. Whereas the two toxins displayed identical cytotoxic potencies, TcdB_NAP1_ induced a stronger proinflammatory response than TcdB_NAP1V_ as determined in *ex vivo* experiments and animal models. Since immune activation at the level of intestinal mucosa is a hallmark of C. difficile-induced infections, we propose that the panel of substrates targeted by TcdB is a determining factor in the pathogenesis of this pathogen and in the differential virulence potential seen among C. difficile strains.

## INTRODUCTION

*C*lostridium difficile, a Gram-positive spore-forming anaerobe, is the leading cause of antibiotic-associated diarrhea in hospitalized patients (1). Antibiotic treatment modifies the balance of commensal microbiota, allowing C. difficile to extensively colonize the gut. The resulting C. difficile infection (CDI) leads to a variety of clinical outcomes that range from mild diarrhea to potentially fatal pseudomembranous colitis (2).

The main virulence factors associated with CDI are two large exotoxins, TcdA and TcdB. The toxins are encoded by the *tcdA* and *tcdB* genes, respectively, which are located in a 19.6-kb pathogenicity locus (PaLoc) together with the *tcdE* (holin-like), *tcdC* (putative negative regulator), and *tcdR* (sigma factor) genes (3, 4). The toxins glucosylate small GTPases (5), and their combined action results in colonic tissue inflammation and massive colonic fluid secretion (2). In cell cultures treated with C. difficile toxins, monoglucosylation of RhoA, Rac1, and Cdc42 disrupts the actin cytoskeleton and causes an arborizing cytopathic effect (CPE) (5).

TcdB is a 270-kDa cytotoxin, and its mechanism of action involves host cell receptor binding (6), uptake by endocytosis (7), pH-dependent pore formation (8), translocation across the endosomal membrane (9), host factor-dependent autoprocessing (10), and release of the glucosyltransferase domain (GTD) into the host cell (11). The C-terminal domain of the holotoxin contains a number of short, homologous regions with combined repetitive oligopeptides (CROPs) and is thought to be important for binding host cell receptor(s) (11). The middle part of the toxin represents the translocation region with autoprocessing activity mediated by an autoprotease domain (9). The GTD located in the N-terminal region is composed of a catalytic and a substrate recognition subdomain; this region is responsible for the cytopathic activity in the host cell cytosol (12).

C. difficile strains producing a variant TcdB have been previously reported, mainly in TcdA-negative strains (13–15). In cultured cells, these TcdB variants induce a CPE characterized by the collapse of the actin cytoskeleton with complete rounding of the cell body and detachment from the surface in contrast to the classic arborizing effect (13). This variant CPE is due to a different pattern of glucosylated GTPases since classic TcdB modifies RhoA, Rac, and Cdc42 whereas variant TcdB targets Rac, Cdc42, Rap, Ral, and R-Ras (5, 13, 14, 16). Furthermore, variations based on the PaLoc sequence have classified these groups of strains in separate toxinotypes (17).

The epidemic NAP1/RT027 C. difficile strains have rapidly spread and have been responsible for epidemic outbreaks worldwide (18, 19). Among the factors that have been proposed to contribute to the increased virulence of these strains are resistance to fluoroquinolones, higher sporulation capacity, and increased production of toxins (20–22). It has been demonstrated that TcdB from epidemic NAP1/RT027 strains possesses an increased cytotoxic capacity on different cell types due to a more efficient autoprocessing activity, which would result in a more rapid release of the enzymatic domain into the cytosol (23). These results indicate that altered TcdB activity could be an additional important factor for the increased pathogenesis of NAP1 strains.

In this work, we describe a C. difficile NAP1 strain from the hypervirulent clade 2 carrying a variant TcdB (TcdB_NAP1V_). In contrast to TcdB from the classic NAP1/RT027 strain, TcdB_NAP1V_ does not glucosylate Rho and partially targets Cdc42. Whereas the cytopathic potency of this TcdB_NAP1V_ is similar to that of TcdB purified from classic NAP1 strains, it induces a significantly lower quantity of proinflammatory mediators in the ligated loop model, suggesting that the panel of glucosylated small GTPases determines the biological outcome induced by C. difficile toxins.

## MATERIALS AND METHODS

### Isolation and characterization of C. difficile strains and fluoroquinolone resistance.

The NAP1 strains were isolated from stool samples according to the protocols previously described (24). Fragments of *tcdA*, *tcdB*, *cdtB*, and *tcdC* were amplified by PCR using primers and conditions previously reported (25, 26). MICs of ciprofloxacin, moxifloxacin, and levofloxacin were determined using agar dilution according to guidelines of the Clinical and Laboratory Standards Institute (CLSI; M11-A7). Resistance breakpoints were >4 μg · ml^−1^. Mutations in the fluoroquinolone resistance-determining region of *gyrA* and *gyrB* and in the *tcdC* genes were identified using Artemis (27) and BLAST tools.

### PFGE typing.

The pulsed-field gel electrophoresis (PFGE) procedure was derived from published protocols (28, 29). Bacteria from 6- to 8-h cultures in brain heart infusion (BHI) were disrupted in lysis buffer. Agarose plugs were prepared by mixing equal volumes of bacterial suspensions and SeaKem Gold agarose (Lonza) in Tris-EDTA (TE) buffer containing SDS. After overnight digestion with SmaI (Roche), DNA fragments were separated on 1% agarose (Bio-Rad) gels. Images were analyzed with the BioNumerics software, v5.1 (Applied Maths), and the patterns were compared to those deposited in the database of the National Microbiology Laboratory, Public Health Agency of Canada (Michael R. Mulvey).

### PCR-restriction fragment length polymorphism (RFLP) analysis and ribotyping.

For toxinotyping, C. difficile VPI 10463 was used as a control according to the published protocols (30). For ribotyping, primer sequences and reaction conditions were taken from the work of Bidet et al. (31).

### Whole-genome sequencing, MLST, and PaLoc/TcdB comparison.

Whole-genome sequences were obtained using multiplexed paired-end libraries and the sequencing-by-synthesis HiSeq platform (Illumina). Reads were assembled using Velvet (32), contigs of >300 bp were scaffolded with SSPACE (33), and gaps were filled using GapFiller (34). The resulting scaffolds were ordered using Mauve (35) and the genomes of reference strain R20291 (NAP1/RT027/ST01) or M68 (NAP9/RT017/ST37). For automatic annotation, we used Prokka (36) and custom C. difficile databases. For core genome multialignment, variant calling, and core genome phylogeny, we used the Harvest suite (37) and FigTree (http://tree.bio.ed.ac.uk/software/figtree/). For multilocus sequence typing (MLST), we used the MLST 1.7 tool maintained by the Center for Genomic Epidemiology at the Danish Technical University (38). PaLoc and TcdB sequences were extracted manually and aligned with MAFFT (39) or MUSCLE (40). For these sequences, phylogenetic tree estimation through maximum likelihood was done using Fasttree (41). TcdB recombination was detected using DualBrothers (42).

### Quantitation of secreted toxins.

The NAP1_V_ and NAP1 strains were grown in TYT broth (3% Bacto tryptose, 2% yeast extract, and 0.1% thioglycolate, pH 6.8) for 24 h, as described previously (29). Decimal dilutions of these supernatants were added to HeLa cell monolayers. The cells were monitored for appearance of CPE. Specific TcdB antiserum (TechLab) was used to neutralize the effect of the toxin. Nontoxigenic C. difficile ATCC 700057 was used as a negative control. Cytotoxicity was expressed as the inverse of the dilution of the supernatants that caused 50% cell rounding in the monolayers (CPE_50_). The amount of toxins was quantified by Western blotting, for which the proteins from bacterium-free supernatants at 24 h were concentrated by methanol-chloroform precipitation (43). Proteins were separated in SDS-PAGE gels and electrotransferred to polyvinylidene difluoride (PVDF) membranes. The membranes were probed with monoclonal anti-TcdA (TTC8) or anti-TcdB (2CV) antibody (tgcBIOMICS) (43). Chemiluminescent signals emitted by a goat anti-mouse IgG-horseradish peroxidase conjugate (Invitrogen) in the presence of the Lumi-Light Plus Western blotting substrate (Roche) were recorded with a ChemiDoc XRS documentation system (Bio-Rad). Transcripts of *tcdA* and *tcdB* were quantified by real-time quantitative PCR (qRT-PCR) as described previously (44). The amplification followed conditions previously reported (44). The relative expression of genes was calculated by the threshold cycle (ΔΔ*C_T_*) method using *rpoA* transcript as the endogenous control (45).

### Toxin purification.

TcdB proteins were obtained from supernatants of NAP1 strains grown in a dialysis system culture and purified as described previously (46). The purity of the toxins was determined by SDS-PAGE and mass spectrometry which indicated the presence of peptides derived from TcdB only and not TcdA (data not shown).

### Cytopathic effect produced by NAP1 toxins.

Confluent 3T3 fibroblasts, Vero cells, and HeLa cells grown in 12-mm glass slides were intoxicated with 0.2 nM TcdB_NAP1_ and TcdB_NAP1V_. The cells were immobilized and fixed according to previously described protocols (47). The CPE was evaluated by phase-contrast, fluorescence, and scanning electron microscopy as indicated in the figure legends.

### *In vitro* glycosyltransferase activity.

The TcdB ability to glycosylate different monomeric GTPases was examined through a radioactivity assay, as previously described (48, 49), and Western blot assays. Briefly, for the radioactive test, UDP-[^14^C]glucose (250 mCi/mmol; PerkinElmer), GTD, each recombinant GTPase–glutathione *S*-transferase (GST), and each TcdB were mixed in a reaction buffer. After 1 h of incubation at 37°C, the proteins were separated by SDS-PAGE. Glycosylation of GTPase was analyzed by phosphorimaging. For graphical representation, band density was measured with ImageQuant TL. For the Western blotting, the same reactions and conditions were used and assays were performed using UDP-glucose (Sigma). After protein separation by SDS-PAGE, the proteins were transferred to PVDF membranes. The GTPase was detected with monoclonal anti-RhoA antibody (Abcam; ab54835) by Western blotting. The control RhoA-GST proteins were stained with Coomassie blue.

### RhoA, Rac1, and Cdc42 GTPase activation assays.

The TcdB ability to inactivate GTPases was determined on confluent 3T3 fibroblasts grown in Dulbecco modified Eagle medium (DMEM) supplemented with 5% fetal bovine serum (FBS) (Sigma). For the pulldown steps, GTP-RhoA was precipitated with GST-tagged Rho binding domain (RBD) and GTP-Rac1 and GTP-Cdc42 were precipitated with GST-p21 binding domain (PBD). Confluent 3T3 fibroblasts cultured in 6-well plates were intoxicated with 0.2 nM TcdB of NAP1_V_ and NAP1 strains under the conditions indicated in the figure legends. After the intoxication, the cells were treated as previously described (47). Briefly, cells were washed with phosphate-buffered saline (PBS) and lysed with precipitation buffer. Lysates were centrifuged and incubated with Rho binding domain (RBD) of the human Rhotekin protein, which had been expressed as a GST fusion protein (RBD-GST), or Rac/Cdc42 (p21) binding domain (PBD) of the human p21-activated kinase 1 protein, which had been expressed as a GST fusion protein (PBD-GST). Active proteins were pulled down by centrifugation, resolved by SDS-PAGE, and transferred to PVDF membranes. GTPases were detected using anti-RhoA (Abcam; ab54835), anti-Rac1 (Abcam; ab33186), or anti-Cdc42 (Abcam; ab41429) antibody by Western blotting. For detection of RhoA glucosylation using the monoclonal antibody, HeLa cells, Vero cells, and 3T3 fibroblasts were intoxicated with 0.2 nM TcdB from NAP1 or NAP1_V_ strains for 6 and 24 h. After intoxication, cells were lysed in 2% SDS and 20 μg of each lysate was separated by 10% SDS-PAGE, electrotransferred to PVDF membranes, and probed with the anti-RhoA monoclonal antibody.

### Structural analysis and TcdB GTD modeling.

The homology models were made using Modeler and Chimera bioinformatics tools, as described previously (49), based on the *tcdB* sequences of NAP1, NAP1_V_, and VPI 10463 strains. Adjustments to the multiple-sequence alignment constructed by using ClustalW (50) were made based on the structure-based alignment performed by superimposing the structures of TcdB proteins.

### Kinetics of CPE induced by toxins.

Confluent HeLa cells were intoxicated with 10 pM TcdB_NAP1_ and TcdB_NAP1V_. The percentage of round cells in each well was evaluated every hour for a period of 12 h and then at 24 h.

### Determination of TNF-α induction.

Confluent RAW 264.7 cells were intoxicated with 0.5 nM TcdB_NAP1_ and TcdB_NAP1V_ for 6 h. The concentration of tumor necrosis factor alpha (TNF-α) in the supernatants was determined by commercial enzyme-linked immunosorbent assay (ELISA) according to the instructions of the manufacturer (R&D Systems).

### Murine ileal loop model.

Animal experimental procedures were approved (CICUA-38-14) by the University of Costa Rica Animal Care and Use Committee. Male Swiss mice of 20 to 25 g were subjected to fasting overnight and anesthetized with ketamine (60 mg/kg of body weight) and xylazine (5 mg/kg) (Eremer Pharma). Through a midline laparotomy, an ileal loop was ligated, and 10 μg of each toxin or the corresponding control solution was injected. Mice were sacrificed 4 h after inoculation, and the length and weight of the intestinal loops were recorded (51). The neutrophil accumulation in homogenized ileal tissue was evaluated through determination of myeloperoxidase (MPO) activity with the *o*-dianisidine dihydrochloride (Sigma) and H_2_O_2_ assay (52). The concentrations of the proinflammatory cytokines interleukin-1β (IL-1β), IL-6, and TNF-α in ileal tissue homogenates were determined by commercial ELISA according to the instructions of the manufacturer (R&D Systems).

### Nucleotide sequence accession numbers.

All reads were deposited at the European Nucleotide Archive in study PRJEB5034 under the run accession numbers ERR467598 (LIBA-5784), ERR467603 (LIBA-6277), ERR467599 (LIBA-5785), ERR467582 (LIBA-5757), and ERR467583 (LIBA-5758).

## RESULTS

### A NAP1 strain inducing a variant cytopathic effect.

**FIG 1:**
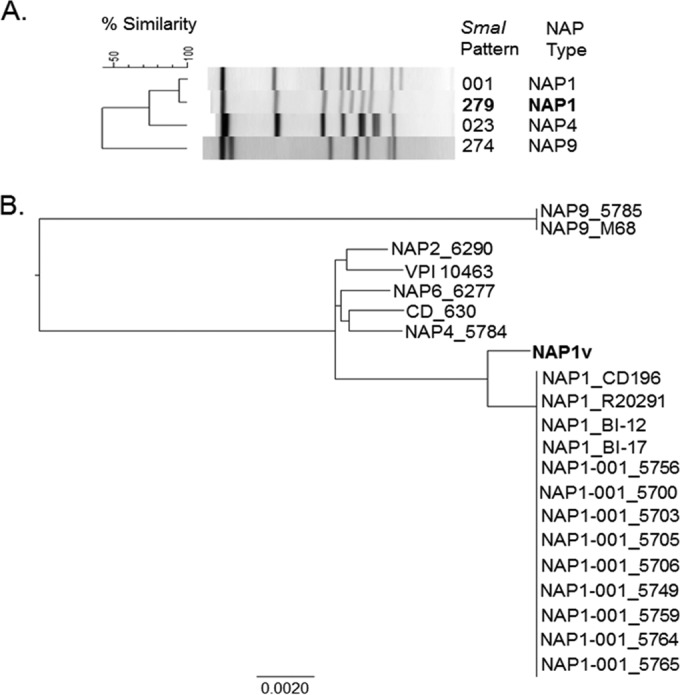
PFGE and core genome-based analysis of the phylogenetic relatedness of NAP1 strains analyzed. (A) Two different SmaI macrorestriction patterns were detected, 001 and 279. The variant NAP1 strain was allocated to the latter group, and thus, we named it NAP1_V_. NAP4 (TcdA^+^ TcdB^+^) and NAP9 (TcdA^−^ TcdB^+^) strains were included in the dendrogram for comparative purposes. (B) A phylogenetic reconstruction based on core SNPs revealed that the NAP1_V_ strain was more closely related to NAP1 reference strains and clinical isolates than to contemporary NAP2, NAP4, NAP6, and NAP9 clinical isolates (four-digit identifiers after the PFGE pattern) and to CD_630 and VPI 10463 strains. The genomes of reference NAP1/RT027 (CD196, R20291, B1-12, and BI17), NAP9/RT017 (M68), CD_630, and VPI 10463 strains were included in the analysis to validate the results of the PFGE typing method.

In a preliminary study performed on a collection of clinical isolates from tertiary care hospitals, the presence of the NAP1 genotype was reported (24). Among 33 NAP1 isolates analyzed, we found a strain whose supernatant induced a cytopathic effect (CPE) different from the classic arborizing CPE observed for the other NAP1 strains (data not shown). PFGE analysis indicated that the SmaI macrorestriction pattern of this particular variant strain was 279, while the pattern of the other NAP1 strains was 001 (Fig. 1A). This strain, here designated NAP1_V_, was analyzed by whole-genome sequencing and comparative genome analyses. A phylogenetic reconstruction based on core single nucleotide polymorphisms (SNPs) revealed that NAP1_V_ is more closely related to historical and epidemic NAP1/RT027/ST01 strains than to TcdA-negative NAP9/RT017/ST37 strains with genes encoding variant TcdB (53) (Fig. 1B). This relationship to the clade 2 of hypervirulent lineages postulated by Griffiths et al. (54) was confirmed through ribotyping and MLST, as both the NAP1_V_ (RT019/ST67) and the NAP1 (RT027/ST01) strains belong to this clade (54). In agreement with this finding, the NAP1_V_ strain carries the *tcdA*, *tcdB*, and *cdtB* genes and presents an 18-bp deletion and a single-base-pair deletion at position 117 in *tcdC*, characteristic of NAP1/RT027 strains. On the other hand, NAP1_V_ was not resistant to fluoroquinolones and did not present the amino acid transition from Thr82 (ACT) to Ile (ATT) in GyrA as observed in classic NAP1/RT027 strains (55).

**FIG 2:**
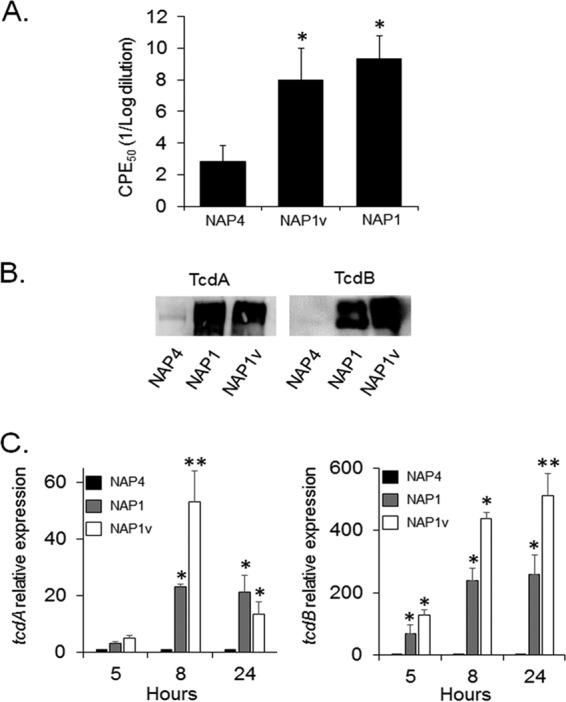
The NAP1_V_ strain produces increased amounts of TcdA and TcdB. (A) Twenty-four-hour bacterium-free supernatants were titrated by 10-fold dilutions on HeLa cell monolayers. Twenty-four hours after inoculation with the indicated supernatant, the dilution inducing a cytopathic effect (CPE) in 50% of the cells was calculated by visual examination under the microscope. Each bar represents the means ± standard deviations of CPE_50_ from three replicates. *, *P* < 0.05 (one-way Kruskal-Wallis test followed by Mann-Whitney U test). (B) Proteins from bacterium-free supernatants were precipitated and separated by 7.5% SDS-PAGE. Proteins were electrotransferred to PVDF membranes and probed with monoclonal antibodies to TcdA and TcdB. (C) Total RNA was prepared from the indicated strains at 5, 8, and 24 h during the growth curve. RNA was retrotranscribed, and cDNA was quantified by RT-PCR using primers specific for *tcdA* and *tcdB*. Results displayed represent the means ± standard deviations from three independent experiments. *, *P* < 0.05 compared to NAP4; **, *P* < 0.05 compared to NAP1 (one-way analysis of variance with Bonferroni's correction).

The levels of secreted TcdA and TcdB and the expression of *tcdA* and *tcdB* transcripts were measured to determine whether the NAP1_V_ strain produces increased amounts of toxin relative to the classical epidemic NAP1/RT027 strain. Titration of toxin activity in bacterium-free supernatants indicated that the NAP1 and NAP1_V_ strains induced similar CPE_50_ titers (Fig. 2A), and in agreement, the levels of secreted toxins were similar for the two strains (Fig. 2B). *tcdA* and *tcdB* mRNAs were quantified by real-time quantitative PCR. The level of both transcripts was significantly higher in both the NAP1 and NAP1_V_ strains than in control strains at all times tested (Fig. 2C). Interestingly, the NAP1_V_ strain produces even more toxin transcripts than does the NAP1 counterpart, a detail that should be considered in future experiments dealing with the regulation of these genes. Altogether, these results demonstrate that the NAP1_V_ strain is closely related to the epidemic NAP1/RT027 strains but displays distinctive genotypic and phenotypic characteristics associated with TcdB that we further explored.

### TcdB_NAP1V_ induces a variant CPE related to a distinct GTPase glucosylation pattern.

**FIG 3:**
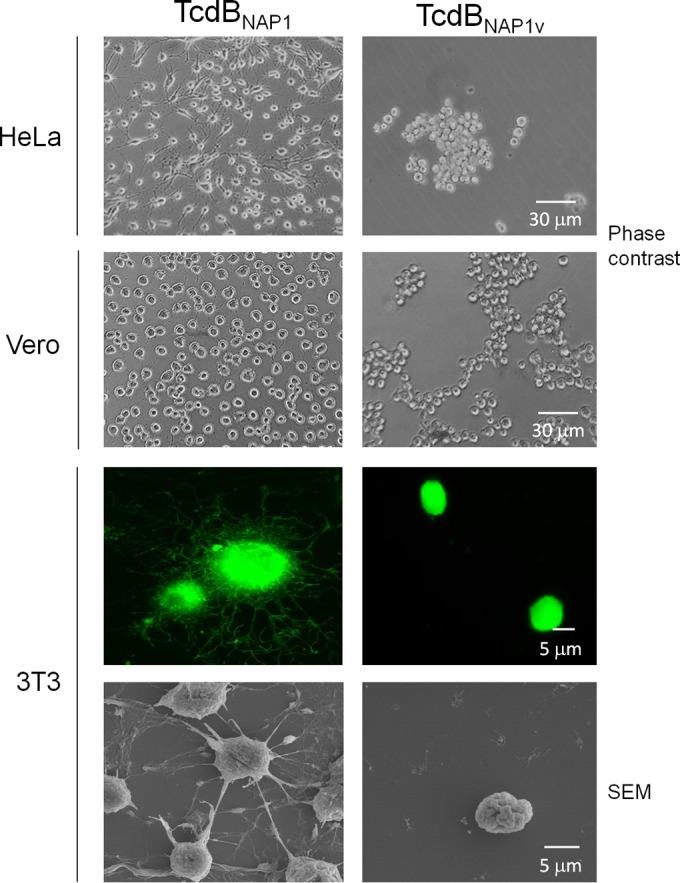
The NAP1_V_ strain induces a variant CPE. HeLa cells, Vero cells, and 3T3 fibroblasts were treated with TcdB_NAP1_ and TcdB_NAP1V_. Cells were treated until a CPE was achieved in 100% of the cells. Images obtained by phase-contrast microscopy show rounding as well as detachment of the cells caused only by TcdB_NAP1V_. In order to see actin cytoskeleton modifications, fibroblasts were stained with fluorescein isothiocyanate-phalloidin. TcdB_NAP1_-treated cells show a classical arborizing effect. TcdB_NAP1V_-treated cells that had not been detached were fixed and show cell rounding without an arborizing effect. Cells were visualized with a Nikon Eclipse 80i fluorescence microscope. Effects on cells induced by the toxins were visualized by scanning electron microscopy (SEM) using an S-3700N (Hitachi) electron microscope.

To analyze the cytopathic characteristics of toxin B from NAP1_V_ (TcdB_NAP1V_) and compare the toxin with those from a classic NAP1 strain (TcdB_NAP1_), both toxins were purified. After intoxication of HeLa cells, Vero cells, and 3T3 fibroblasts with TcdB_NAP1_, the classical arborizing CPE was observed (Fig. 3). In contrast, TcdB_NAP1V_ induced cell rounding and detachment but no protrusions or arborizing effects (Fig. 3). Hence, TcdB_NAP1V_ was responsible for the variant CPE produced by NAP1_V_ supernatants.

**FIG 4:**
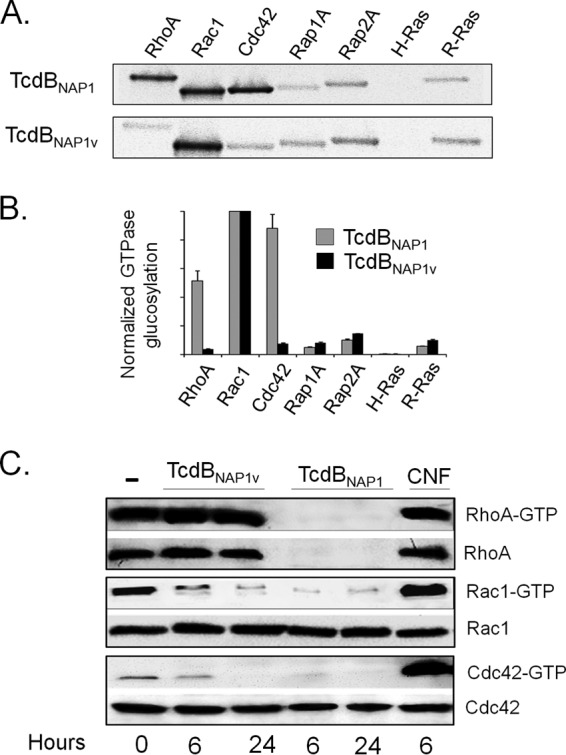
The NAP1_V_ strain does not glucosylate RhoA. (A) TcdB_NAP1_ and TcdB_NAP1V_ were tested for their ability to glycosylate a panel of recombinant GTPases using UDP-[^14^C]glucose as a cosubstrate. Labeled bands were detected by phosphorimaging analysis. (B) The band intensities of the GTPase glycosylation were quantified by densitometry. Each experiment was normalized to Rac1 signal. Means ± standard deviations from three independent experiments are shown. (C) Effect of TcdB_NAP1_ and TcdB_NAP1V_ on the activation state of small GTPases. 3T3 fibroblasts were intoxicated with TcdB_NAP1_ and TcdB_NAP1V_ for the indicated times. After treatment, cells were lysed. One part of the lysates was used as a control for total amount of GTPases, and the other one was incubated with PBD-GST or RBD-GST-Sepharose beads. Active proteins were pulled down and analyzed by Western blotting. GTPases were detected using anti-RhoA, anti-Rac, and anti-Cdc42, respectively. Cells treated with TcdB_NAP1_ show inactivation of RhoA, whereas cells intoxicated with TcdB_NAP1V_ do not. Cytotoxic necrotizing factor 1 (CNF) from Escherichia coli was used as a positive control for GTPase activation. Negative-control cells were left untreated.

Next, we determined the glucosylation pattern of TcdB_NAP1_ and TcdB_NAP1V_ using a panel of small GTPases and a radioactive *in vitro* assay. TcdB_NAP1_ modified a panel of substrates characteristic of classic TcdB proteins, with RhoA, Rac1, and Cdc42 being readily glucosylated (Fig. 4A). Interestingly, we observed modification to a lesser extent of Rap1, Rap2, and R-Ras, which has not been reported previously for classic TcdB proteins inducing arborizing CPE. On the other hand, TcdB_NAP1V_ glucosylated Rac1, but the glucosylation of RhoA and Cdc42 was significantly diminished (Fig. 4B). As with TcdB_NAP1_, TcdB_NAP1V_ was able to glucosylate Rap1, Rap2, and R-Ras at low levels.

**FIG 5:**
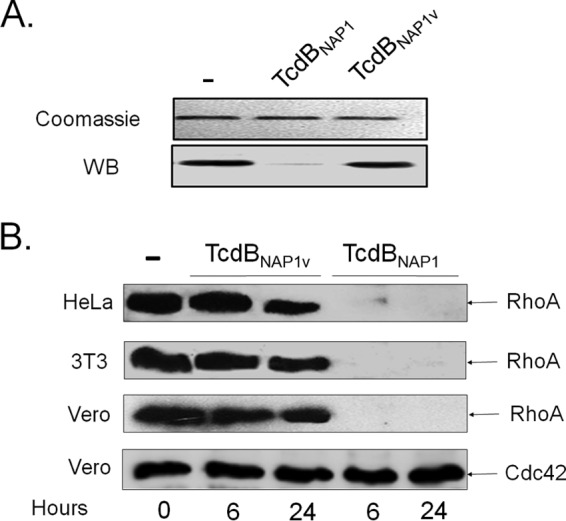
A monoclonal antibody to RhoA detects modification of this small GTPase by TcdB_NAP1_ but not by TcdB_NAP1V_. (A) Recombinant purified RhoA was incubated with TcdB_NAP1_ and TcdB_NAP1V_ in the presence of UDP-glucose. The preparations were separated by SDS-PAGE and detected by Coomassie blue staining. Parallel samples were transferred to PVDF membranes and developed by Western blotting (WB) using the monoclonal antibody to RhoA. (B) HeLa cells, 3T3 fibroblasts, and Vero cells were treated for the indicated times with TcdB_NAP1_ or TcdB_NAP1V_. Cell lysates of treated cells and nontreated control cells were separated by SDS-PAGE, transferred to PVDF membranes, and revealed with the monoclonal antibody to RhoA by Western blotting. As a loading control, membranes were also revealed with a monoclonal antibody to Cdc42.

To confirm the panel of substrates modified by the toxins, we monitored the *ex vivo* glucosylation of RhoA, Rac1, and Cdc42 after intoxication of cultured cells by pulldown assays. When 3T3 cells were incubated with TcdB_NAP1_, Rho-GTP was undetectable at 6 h (Fig. 4C). In contrast, Rho-GTP was detected in cells intoxicated with TcdB_NAP1V_ for up to 24 h, confirming the lack of modification of this small GTPase in the *in vitro* assay (Fig. 4C). Both TcdB proteins inactivated Rac1 after 6 and 24 h of treatment, again confirming the results of the *in vitro* glucosylation assay (Fig. 4C). Additionally, the level of Cdc42-GTP exhibited a significant and consistent decrease at 6 h after intoxication with TcdB_NAP1_ and Cdc42-GTP completely disappeared after 24 h of treatment (Fig. 4C). However, TcdB_NAP1V_ was able to decrease the level of Cdc42-GTP only after 24 h of intoxication, indicating that this small GTPase is not a preferred substrate of this variant TcdB (Fig. 4C). Interestingly, no signal was detected in the control for total Rho (loading control) in cells treated with TcdB_NAP1_, indicating either that the protein is degraded after glucosylation or that, alternatively, the antibody to Rho that we used in this work does not recognize the glucosylated isoform (Fig. 4C). To distinguish between these two possibilities, recombinant Rho was incubated with either TcdB_NAP1_ or TcdB_NAP1V_ in the presence of UDP-glucose. Rho was detected by Coomassie blue staining after treatment with both toxins but was not detected by Western blotting after treatment with TcdB_NAP1_ (Fig. 5A). This result indicates that the monoclonal antibody used does not interact with glucosylated Rho and confirms the fact that TcdB_NAP1V_ does not modify this small GTPase. Thus, this monoclonal antibody represents a valuable tool to monitor Rho modification by large clostridial cytotoxins. To further explore this concept, different cell lines (HeLa cells, 3T3 fibroblasts, and Vero cells) were intoxicated for 6 and 24 h with either TcdB_NAP1_ or TcdB_NAP1V_. RhoA was detected only in lysates prepared from cells intoxicated with TcdB_NAP1V_ (Fig. 5B), confirming that the ability to target this small GTPase is the main difference at the level of substrates between the two toxins.

### TcdB_NAP1V_ sequence combines the enzymatic domain of variant toxins with the receptor-binding domain of TcdB from the hypervirulent clade 2.

**FIG 6:**
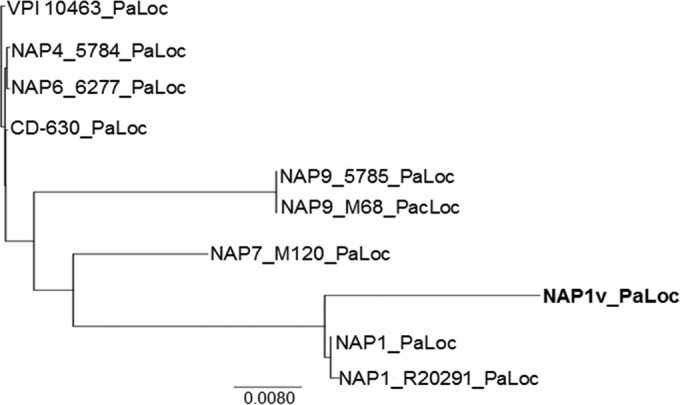
Phylogenetic relationship of the pathogenicity locus (PaLoc) of NAP1_V_ and NAP1 strains to that of reference strains (VPI 10463, CD_630, NAP7/RT078_M120, NAP9/RT017_M68, and NAP1/RT027_R20291) and clinical isolates (NAP4, NAP6, and NAP9). The PaLoc sequence of NAP1_V_ clustered together with those of clinical (NAP1-001_5768) and reference NAP1/027 (R20291) strains rather than with sequences from TcdA^−^ TcdB^+^ variant strains (NAP9/017).

**FIG 7:**
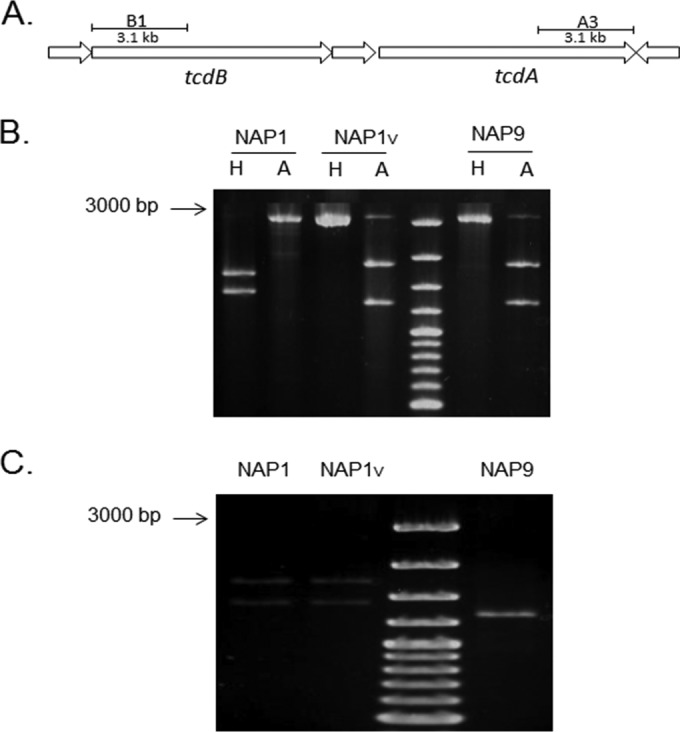
Toxinotyping of NAP1 strains. The polymorphisms obtained from the B1 and A3 regions of the *tcdA* and *tcdB* genes were analyzed by digestion with AccI (A) and HindIII (H) restriction enzymes. (A) Representation of the amplified regions. (B) The restriction polymorphisms of the *tcdB* fragments from the NAP1_V_ (toxinotype XXII) and the *tcdA*-negative *tcdB*^+^ NAP9 (toxinotype VIII) strains are indistinguishable and different from the corresponding pattern from the NAP1 strain. (C) The NAP1_V_ and NAP1 (toxinotype III) strains have the same restriction pattern of the *tcdA* fragment.

Since TcdB_NAP1V_ clearly presents different phenotypic behavior than TcdB_NAP1_, we focused on differences at the sequence level. Phylogenetic analysis indicates that the PaLoc of the NAP1_V_ strain is more closely related to that of classic NAP1/RT027 strains than to TcdA-negative strains carrying variant TcdB proteins (Fig. 6). Next, we determined the toxinotype of the NAP1_V_ strain. The *tcdB* polymorphisms of the B1 fragment (containing the coding region for the TcdB glucosyltransferase domain) (Fig. 7A) from NAP1_V_ were identical to those of TcdA-negative strain NAP9/RT017 and different from that of NAP1/RT027 (Fig. 7B). The restriction patterns for *tcdA* were the same for the two strains (Fig. 7C). Thus, the toxinotype of the NAP1_V_ strain (toxinotype XXIII) is not the classic one found in NAP1 strains (toxinotype III) and rather coincides with the toxinotype present in TcdA-positive strains carrying variant TcdB proteins.

**FIG 8:**
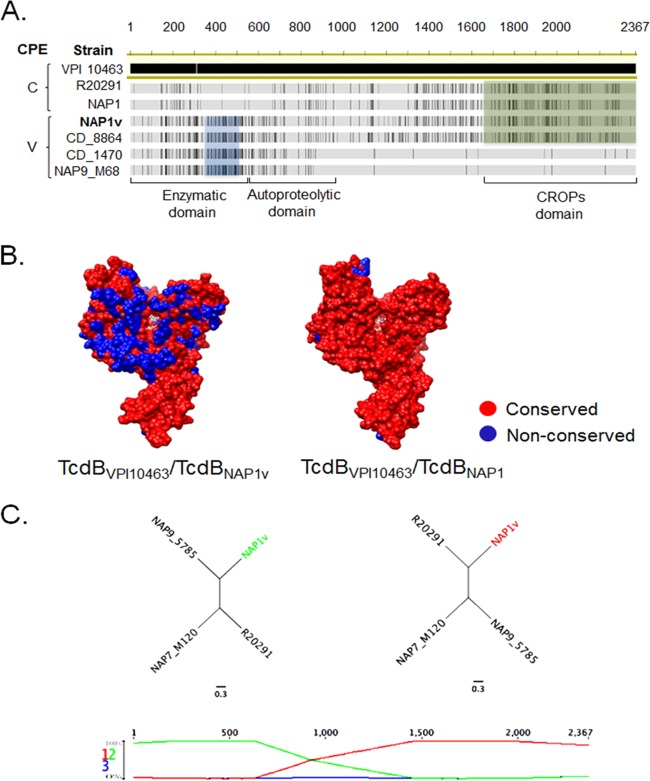
TcdB_NAP1V_ shares with TcdB_NAP1_ the receptor-binding domain but not the enzymatic domain. (A) Sequence alignment of (i) variant toxins B from NAP9_M68, CD_1470, and CD_8864 reference strains inducing a variant (V) CPE; (ii) TcdB_NAP1_ from a clinical isolate and a reference epidemic NAP1/RT027 strain (R20291) inducing a classic (C) CPE; and (iii) TcdB_NAP1V_. Black lines represent disagreements in the sequence of TcdB_VPI10463_, which was selected as a reference for the alignment. The blue box highlights a distinct glucosyltransferase region shared between NAP1_V_ and other variant strains. The green box shows sequence stretches in the repetitive CROPs domains shared between TcdB_NAP1_, TcdB_R20291_, and TcdB_NAP1V_. (B) Comparison of the TcdB_NAP1_ and TcdB_NAP1V_ sequences in the context of the TcdB GTD structure (PDB 2BVM, VPI 10463 sequence). Sequence conservation on the putative GTPase-binding face compared to the GTD from C. difficile VPI 10463 with NAP1 and NAP1_V_ TcdB GTD structures is shown (red, conserved; blue, not conserved). UDP-glucose is depicted in white in the GTD active site. (C) Resulting recombination detection graphs using TcdB sequences from strains NAP9 (M68, RT017 reference strain), R20291 (epidemic NAP1/RT027 reference strain), NAP7 (epidemic M120, RT078 reference strain), and NAP1_V_. Signs of possible recombination events are represented as changes in the topology graphs (first row) that appear at the most probable topology between the segments. The cross of the topological lines (green and red lines) indicates recombination breakpoints. Resulting trees are compatible with a scenario in which TcdB_NAP1V_ emerged through recombination of *tcdB* sequences from NAP9 and NAP1 strains.

A detailed analysis and comparison of the sequences from the different TcdB proteins indicates that the primary sequence of the glucosyltransferase domain of TcdB_NAP1V_ is more closely related to the corresponding region of TcdB proteins inducing a variant CPE than to that of TcdB proteins inducing a classic arborizing CPE (Fig. 8A). Indeed, the identity in the first 546 amino acid residues between TcdB_NAP1V_ and TcdB_NAP9_ is 100%, whereas that between TcdB_NAP1V_ and TcdB_NAP1_ is 80%. Furthermore, the identity in the substrate specificity domain (amino acids 365 to 516) between TcdB_NAP1V_ and TcdB_NAP1_ is only 62%. The glucosyltransferase domain (GTD) sequences of TcdB_NAP1V_ and TcdB_NAP1_ were analyzed in the context of the VPI 10463 reference strain (identical to the 630 reference strain). Although the core residues of the GTDs are conserved between TcdB_NAP1V_ and TcdB_VPI10463_, the surface residues are divergent (Fig. 8B). These divergent residues are predicted to be involved in the substrate affinity of the GTD. In contrast, the GTDs of TcdB_NAP1_ and TcdB_VPI10463_ are very similar (Fig. 8B). The CROPs domain of TcdB_NAP1V_ is highly similar to the corresponding region of TcdB proteins from classic NAP1 strains (Fig. 8A). The identity in this region (amino acids 1645 to 2366) between TcdB_NAP1V_ and TcdB_NAP1_ is 99%.

These data indicate the possibility of a recombination event that led to the sequence encoding TcdB_NAP1V_. To explore this, we applied a Bayesian approach to infer changes in tree topologies and evolutionary rates using TcdB sequences from strains NAP1_V_, R20291 (NAP1/RT027), M120 (NAP7/RT078), and NAP9/RT017. Figure 8C shows the dominant tree topology within each partition of the alignment, with NAP1_V_ and NAP9/RT017 being the closest neighbors with 100% probability in the first 635 residues of the alignment and NAP1_V_ and R20291 (NAP1/RT027) showing the same result from residue 1465 onward. These topologies imply a possible recombination event between TcdB proteins from NAP1/RT027 and NAP9/RT017 strains.

### TcdB_NAP1V_ and TcdB_NAP1_ have similar cytopathic potencies but exert different biological effects.

**FIG 9:**
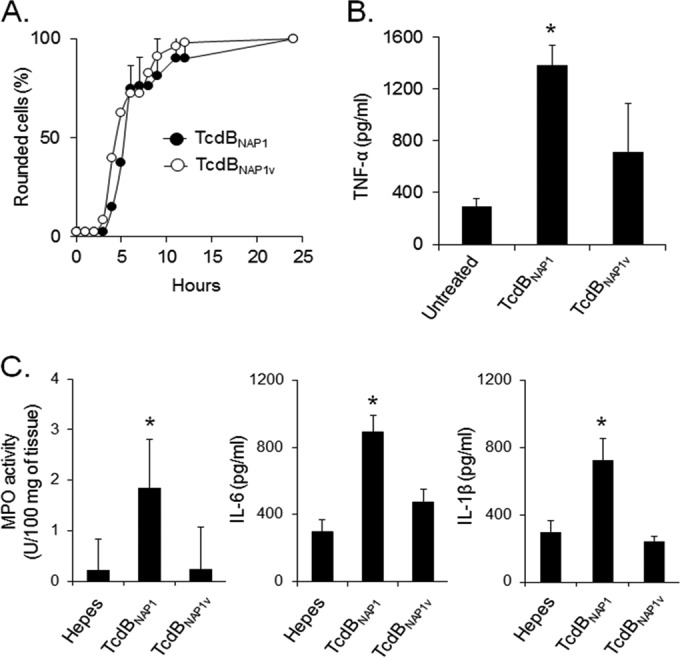
TcdB_NAP1V_ has the same cytotoxic potency as TcdB_NAP1_ but induces fewer proinflammatory reactions. (A) HeLa cells were treated with equal concentrations (10 pM) of purified TcdB_NAP1_ and TcdB_NAP1V_. The percentage of cells showing a toxin-induced CPE was calculated at the indicated times. (B) RAW cells were incubated for 6 h with equal concentrations (0.5 nM) of purified TcdB_NAP1_ and TcdB_NAP1V_. After incubation, the amount of TNF-α released in the supernatant was determined by ELISA. Each bar represents the mean ± standard deviation from three independent experiments. (C) Mouse ligated ileal loops were inoculated with 10 μg of purified TcdB_NAP1_ and TcdB_NAP1V_ for 4 h. After treatment, MPO activity and inflammatory cytokine (IL-1β and IL-6) levels were determined. Means ± standard deviations, *n* ≥ 5. *, *P* < 0.05, compared to the groups without asterisk (one-way analysis of variance with Bonferroni's correction).

To assess the cytopathic potency of TcdB_NAP1V_ and TcdB_NAP1_, both toxins were titrated on HeLa cells. The two toxins elicited similar kinetic profiles of cell intoxication, indicating that their cytopathic potencies are similar (Fig. 9A). TcdB_NAP1V_ and TcdB_NAP1_ were also tested for their ability to induce TNF-α production by RAW murine macrophages. Despite the similar cytopathic potencies, the release of this cytokine was statistically higher in cells treated with TcdB_NAP1_ (Fig. 9B). The pathogenic potential of both toxins was assayed in the murine ligated ileal loop model. We measured the concentration of myeloperoxidase (MPO) activity as an indicator of tissue neutrophil infiltration and the levels of IL-1β and IL-6 to indicate immune activation at the ileal tissue level. TcdB_NAP1_ caused a statistically significant increase in MPO activity, whereas TcdB_NAP1V_ elicited a reaction undistinguishable from that of the control (Fig. 9C). The levels of IL-1β and IL-6 were significantly increased by TcdB_NAP1_, and again, TcdB_NAP1V_ was unable to induce any reaction (Fig. 9C).

## DISCUSSION

The increase in rate and severity of CDI has been linked to the emergence and spread of the epidemic NAP1 strain (19, 56). This genotype has acquired several genetic determinants that contribute to its increased virulence; among these, the overproduction of toxins (linked to mutations in the *tcdC* gene), the presence of a binary toxin, and resistance to fluoroquinolones have been considered to play an important role (21, 22, 56). It has been shown that TcdB is essential for C. difficile virulence and that its glucosyltransferase activity is required for activity in an ileal loop model (57, 58). In this context, previous studies have concluded that variations in the sequence of this toxin lead to an augmented cytopathic potency and play a crucial role in the increased virulence of NAP1 strains. This increased cytotoxicity is due to a more efficient delivery of the enzymatic domain to the cytosol (23, 59). However, the role of the substrate pattern of TcdB within the hypervirulent clade 2 has not been analyzed. In the present study, we describe a NAP1 isolate from an outbreak setting that produces a variant TcdB. Our goals were to understand the differences in the CPEs induced by this toxin and the potential role of the panel of modified substrates in the biological effects induced by TcdB proteins secreted by strains from the hypervirulent clade 2 and to elucidate the emergence of this NAP1 variant strain.

Due to the PFGE classification and the particular CPE induced by bacterium-free supernatants derived from the NAP1_V_ strain, we reasoned that this isolate would have phenotypic characteristics associated with important differences at the level of TcdB. The NAP1_V_ strain is, in fact, a toxin-overproducing isolate, harboring deletions in *tcdC* and carrying the binary toxin gene. Nonetheless, it is not resistant to fluoroquinolones, since it does not harbor the typical mutation in *gyrA* found in NAP1 strains (60). Despite the strain belonging to the NAP1/RT027 genotype, the NAP1_V_/RT019 macrorestriction pattern differs from that of the classical NAP1/RT027 strains and its toxinotype differs due to variations within the *tcdB*-encoded N-terminal region. Indeed, the digestion pattern of the amplified B1 fragment coding for the catalytic region of TcdB_NAP1V_ was indistinguishable from the corresponding one presented in TcdA-negative strains. Interestingly, the NAP1_V_ strain belongs to the toxinotype XXII, which has been described in isolates harboring variant TcdB proteins (17, 61).

The CPE induced by TcdB_NAP1V_, characterized by rounding and detachment of intoxicated cells, resembles the effect induced by Clostridium sordellii lethal toxin (TcsL) and C. difficile variant TcdB proteins (13, 62).This effect, referred to as variant CPE, was first reported in C. difficile strains that do not produce TcdA. While it has now been described in a wider range of strains (13, 17, 47), it had not been previously described within the NAP1/RT027 genotype; in all these cases, the variant CPE has been attributed to TcdB (16). The induction of a variant CPE by variant TcdB proteins correlates with substrate profiles that differ from the panel targeted by TcdB from reference strain VPI 10463. Whereas the latter modifies Rho, Rac, and Cdc42, variant TcdB proteins also modify R-Ras, Rap, and Ral (5, 47). A more detailed analysis of the consequences of small GTPase modification indicated that R-Ras glucosylation and transient RhoA activation determine the appearance of a variant CPE since R-Ras glucosylation leads to integrin inactivation, and as a consequence, focal adhesions disassemble, causing detachment (47). In contrast to TcdB from strain VPI 10463, TcdB_NAP1_, which induces a classic arborizing CPE, seems to have an extended substrate pattern since it was able to modify Rap and R-Ras. These additional targeted substrates might have a role in the increased biological effects induced by TcdB_NAP1_ (57, 59). Interestingly, the main difference at the level of substrates between TcdB_NAP1V_ and TcdB_NAP1_ is the modification of RhoA. Thus, the ability to target this small GTPase also seems to play an important role in defining the type of CPE that will be induced, since RhoA-modifying toxins induce an arborizing CPE and non-RhoA-modifying toxins induce a variant CPE (63). In addition, previous studies have shown that Cdc42 is not glycosylated by variant TcdB proteins, but in the case of TcdB_NAP1V_, there is partial modification of this small GTPase. However, a complete glucosylation of Cdc42, as determined by pulldown assays, was detected only after 24 h of intoxication, indicating that glucosylation of this protein is not involved in induction of the variant CPE, which appears in the first few hours after addition of the toxin.

There is a clear correlation between the small GTPases modified and the type of CPE induced by TcdB_NAP1V_ and variant TcdB proteins from TcdA-negative strains. This concordance is in agreement with the primary sequence of the toxins since the GTD of TcdB_NAP1V_ has a high identity to the corresponding domain found in variant TcdB proteins. On the other hand, the autoprocessing domain and the carboxyl-terminal region of TcdB_NAP1V_ are almost identical to the corresponding regions from TcdB_NAP1_. These results, along with sequence comparison, reveal that TcdB_NAP1V_ is a toxin of the classical NAP1/RT027 genotype but with modifications within the enzymatic domain.

Recently, a strain belonging to clade 2, RT244/ST41, was reported to display an increased virulence (62). As a NAP1 strain, RT244/ST41 harbors a binary toxin; however, it does not produce increased amounts of toxins and is fluoroquinolone susceptible. These data indicate that the NAP1_V_/RT019 strain shares more features with the classic NAP1/RT027 than RT244/ST41 and would then be more closely related to the epidemic strain. Interestingly, the strain RT244/ST41 genome also seems to encode a variant TcdB. A comparative and detailed assessment of the virulence potential of these three strains would allow one to determine the relative contribution of factors such as the presence of binary toxin, overproduction of toxins, fluoroquinolone resistance, and type of toxin produced to the increased virulence displayed by members of this clade.

Nosocomial outbreaks caused by TcdA-negative strains have increased in the last decade (53, 64, 65). Interestingly, all these strains have been reported to harbor variant TcdB proteins. This might be an indication that the biological effects induced by classic TcdB proteins differ from those induced by variant TcdB proteins and that TcdA-negative strains compensate for the lack of this toxin by using a TcdB with a different panel of substrates. When we compared the responses to TcdB on the ligated loop model, we could indeed find a significant biological difference between the two toxins. Whereas TcdB_NAP1_ induced an immune activation, TcdB_NAP1V_ induced a much milder and almost undetectable response. Secretion of TNF-α by macrophages has been associated with the glucosyltransferase activity of C. difficile toxins (66), and since TcdB_NAP1V_ and TcdB_NAP1_ have a high degree of identity in the regions determining receptor binding and entrance to the cell and the two toxins have similar cytopathic potencies, we assume that the biological differences detected in our assays are due to a differential panel of substrates glucosylated. Since the main difference in substrates is at the level of RhoA modification, we hypothesize that glucosylation of this small GTPase enhances the proinflammatory response induced by C. difficile toxins. The use of a panel of purified toxins with differing substrate panels like the ones indicated in this article in a wide range of experimental models would allow the dissection of the relevance of the modification of different small GTPases in the outcome of CDI.
